# The perception of hidden curriculum among undergraduate medical students: a qualitative study

**DOI:** 10.1186/s13104-018-3385-7

**Published:** 2018-05-04

**Authors:** Zahra Gardeshi, Mitra Amini, Parisa Nabeiei

**Affiliations:** Clinical Education Research Center, Shiraz University of Medical Sciences, Neshat Street Sina Sadra Hall, Shiraz, Iran

**Keywords:** Hidden curriculum, Medical students, Qualitative study

## Abstract

**Objectives:**

The effect of hidden curriculum on student learning has not been sufficiently recognized in most of the revised curriculums. This study is a qualitative study that measures the students’ perception of hidden curriculum through semi-structured interviews. All of the interviews were recorded and then converted into scripts. These scripts were divided to sentences and phrases and named as units. Units aggregated with similar groups and named as codes, then the similar codes were aggregated into themes.

**Results:**

Four main themes emerged, role modeling, personal attitude and beliefs, hierarchy, social and ethical culture. The results of the present study showed that it is necessary to discuss the hidden curriculum. We are unaware of the hidden curriculum, but even when were are aware of it, we are unwilling to act. Information about issues related to the hidden and informal curriculum, as well as knowing the viewpoints of students is necessary. It seems necessary to provide data to students about the hidden curriculum and encouraging patient centered curriculums early in training, such as integrated curriculum.

## Introduction

Hidden curriculum is defined as a set of unwritten, unofficial and unintended lessons, values and perspectives that students learn in medical school along with more formal aspects of education [1].

The hidden curriculum is a set of stimuli that act at the level of organizational culture [2]. Learning connected with the hidden curriculum may have potential undesirable consequences on trainee educational development [3]. Hidden aspects of the curriculum are important in professional education, which include long periods of contact with the predominant culture [4–6]. The necessity of speaking about the hidden curriculum has been highlighted by medical educators [7–9].

During recent years most medical schools in Iran have changed the undergraduate medical education curriculum [10–14]. However, the effect of the hidden curriculum, as powerful concept in medical education, on students learning, has not been sufficiently recognized in most of the revised curriculum around the world [2, 15, 16]. This may be attributed to the pressure of the formal curriculum [4]. Qualitative studies have been effective in exploring issues linked to the hidden curriculum [3]. In spite of similarities with previous published articles about hidden curriculum around the world, we aimed to explore the perception of hidden curriculum among the students of Shiraz University of Medical Sciences. We believed that each school has its own hidden curriculum model. We used semi-structured interviews for this purpose because semi-structured interviews allow respondents to show their views in their own terms freely. Semi-structured interviews can provide valid and reliable data.

## Main text

### Methods

This study was a qualitative study that measured the students’ perception of the hidden curriculum through semi-structured interviews. 32 students studying in years 1–7 at Shiraz Medical School, southern Iran, were interviewed. The integration between education and health services was done in this school many years ago [17]. We used maximum variation sampling [18].

Our variables included sex, ethnicity, home or dormitory living, marital status and year of study to ensure that maximum variation was obtained. Data gathering was done based on the data saturation concept.

Semi-structured interviews:

Semi-structured interviews were done based on general questions such as following questions:

- What do you think about your educational system in general?
- By which method would necessary knowledge and skills be acquired in your training period?
- What motivates you to study?
- What motivates you to enjoy learning?
- Are you satisfied with your medical training?
- How do you meet your teachers, mentors, and colleagues?
- Do you have role models?

Interviews were done in private rooms with students. The time of each interview was about 30 min.

#### Data analysis

The data were analyzed using inductive content analysis method [19]. The unit of analysis was recorded interviews, each one lasted approximately 45–60 min, then each interview was transcribed; they were analyzed and then were read again to gain a broader view.

According to the units of analysis, the texts were separated into summarized meaning units as words, sentences or sections holding features connected to each other over their content. Then, each summarized unit was abstracted and named with a code. Different codes were matched based on the relationship of underlying meanings, and the same meanings were collected together, which formed themes. Finally, four themes were formed and agreed upon by the researchers.

#### Trustworthiness

The reliability and validity of the questionnaires should be checked in qualitative research [19]. Credibility is to create confidence in clarification of meaning of the data [19]. In this study, complete explanations were used that decreased the probability of losing information. Member check was another technique in which we sent a copy of the transcript with codes to participants and requested them to evaluate researchers’ understanding with their own concepts. Peer check was also done in our study; to do so, units, codes and themes were checked by a researcher familiar with the qualitative research. In order to obtain dependability, in-depth methodological explanation was used to permit the repetition of the study. In transferability, this discusses the extent to which study findings can be appropriate in contexts outside the study condition [20]. Total explanation of the phenomenon under study was delivered to allow readers, thereby allowing them to associate the instances of the phenomenon defined in the research report with those they had observed to appear in their situations.

#### Ethical considerations

Students who participated in the study voluntarily and their names were not mentioned in the scripts.

### Results

Four main themes emerged in this study: role modeling, personal attitude and beliefs, hierarchy, social, and ethnic culture.

#### Role models

This theme was selected when students talked about their relationship with teachers who had influenced their attitude toward teaching and learning. The three most frequently mentioned characteristics of positive role models were politeness, good communication with students in any field, and proficiency in their specific field.

The students reported that most of their teachers were positive role models who had an inspiring and encouraging influence on them. Students who reported their teachers’ role as negative became disinterested in learning.

As one student ascertained:

There is a team of lecturers that I think are excellent. They were really good lecturers, they were accessible and you could communicate with them about anything.

Another student said:

I liked teachers that were enthusiastic about their discipline and had excellent skills related to their specialty. These kinds of teachers motivated me to study hard.

#### Personal attitude and beliefs

A view frequently reported by students in semi-structured interviews was the effect of positive and negative attitude towards medicine.

One of the students said: A positive attitude about being a good physician encouraged me to be a successful student.

Another student argued that: My family insisted that I become a physician. I don’t like medicine myself and I have no motive to study.

#### Hierarchy

This theme was reported specially by students in the clinical period. Some of the students said that they were treated badly in clinical wards for no reason except their inferior position in the ward. There were also numerous contacts between senior nurses, midwives, and medical students.

One of the students said: At the beginning of my rotation in the Coronary Care Unit the senior resident blamed me for not being able to answer questions about the Electro CardioGram(ECG) pattern.

Another student reported: When I went to the labor room, the midwives always said we are tired of teaching students that don’t know anything. Some of the nurses in the wards also gave us a bad time in the wards. In the operating room the paramedical staff always shouted at us “you ruined the sterility”.

Another student said: Medical school is like a military you should respect and obey all the persons that are superior.

#### Social and ethnic culture

This theme was reported approximately in all interviews. Shiraz Medical School is in the south of Iran and many ethnic groups study in this school including “Fars”, “Lor”, and “Turk”. This ethnic and cultural variability has some positive and negative points.

Competition between these groups was reported to be a negative consequence by the students, but the positive impact of this cultural diversity was reported by some of the students. There was a report of cooperative learning activities such as Peer Assisted Learning (PAL) and mentorship in which all the students tried to cooperate with each other and learn from their peers.

One of the students reported: As a team we need to do the whole thing cooperatively. We have a lot of cooperative groups in our medical school such as PAL group, mentorship group, etc.

Another student said: I am not a native student and I have some problems communicating with other students. We have competition and disagreement with native students in various fields.

### Discussion

We are occasionally unconscious about the hidden curriculum, but even when conscious of it we do not do anything about it. The results of our study was similar to results of other published studies all over the world [2, 21, 22]. The results indicated that there were some similarities between countries in some aspects of the hidden curriculum in medical schools.

Regarding the theme of role modeling, it had positive effects on students in most of the cases. In a qualitative study about role modeling the results showed that clinical teachers’ awareness of their own obvious professional characteristics could help generate better teaching and learning experiences [23]. Another study showed that clinical attributes, teaching skills, and personal qualities of clinical teachers are the characteristics that medical students reported for a good role model [24].

Personal attitude and beliefs was another theme in our study and the results were similar to our previous qualitative study about important factors for medical students’ success [25]. In this study, hierarchy was reported to have a negative impact and led to humiliation. The theme hierarchy has been reported in other studies [1, 21]. It seems necessary to cease the silence in the field of hierarchy in medical education and report unprofessional and unethical behavior if observed.

The last theme was about social and ethnic culture. Shiraz Medical School is located in Fars province in the south of Iran. It consists of different social and ethnic groups such as” Fars”, “Lor”, and “Turk”. Also due to the central selection of medical students in Iran all other ethnic groups are also studying medicine at Shiraz Medical School. Cooperation and team activities are mentioned as positive points in this theme. Cultural sensitivity and competition were negative findings. It seems necessary to have extracurricular activities for medical students to engage in teams and work together [26]. Research in the field of curriculum was one of the priorities in medical education field in Eastern Mediterranean Region and Iran [27, 28].

We are unaware of the hidden curriculum, but even when are aware of it, we are unwilling to act. Information about issues related to the hidden and informal curriculum, as well as knowing the viewpoints of students is necessary. It seems necessary to provide data to students about the hidden curriculum and encouraging patient centered curriculums early in training, such as integrated curriculum [29].

## Limitation

The limitation of this study was that the study was done based on interviews and we could not observe the students. The other limitation was that the data was gathered from Shiraz Medical School that may not be a representative of all Iranian medical schools; however, we know that in qualitative research we are not trying to reach external validity.

## Abbreviations

ECG: electro cardio gram
PAL: Peer Assisted Learning
